# DNA G-quadruplex structure participates in regulation of lipid metabolism through acyl-CoA binding protein

**DOI:** 10.1093/nar/gkac527

**Published:** 2022-06-24

**Authors:** Lijun Xiang, Kangkang Niu, Yuling Peng, Xiaojuan Zhang, Xiaoyu Li, Ruoqi Ye, Guoxing Yu, Guojun Ye, Hui Xiang, Qisheng Song, Qili Feng

**Affiliations:** Guangzhou Key Laboratory of Insect Development Regulation and Application Research, Institute of Insect Science and Technology, School of Life Sciences, South China Normal University, Guangzhou 510631, China; Guangzhou Key Laboratory of Insect Development Regulation and Application Research, Institute of Insect Science and Technology, School of Life Sciences, South China Normal University, Guangzhou 510631, China; Guangzhou Key Laboratory of Insect Development Regulation and Application Research, Institute of Insect Science and Technology, School of Life Sciences, South China Normal University, Guangzhou 510631, China; Guangzhou Key Laboratory of Insect Development Regulation and Application Research, Institute of Insect Science and Technology, School of Life Sciences, South China Normal University, Guangzhou 510631, China; Guangzhou Key Laboratory of Insect Development Regulation and Application Research, Institute of Insect Science and Technology, School of Life Sciences, South China Normal University, Guangzhou 510631, China; Guangzhou Key Laboratory of Insect Development Regulation and Application Research, Institute of Insect Science and Technology, School of Life Sciences, South China Normal University, Guangzhou 510631, China; Guangzhou Key Laboratory of Insect Development Regulation and Application Research, Institute of Insect Science and Technology, School of Life Sciences, South China Normal University, Guangzhou 510631, China; Guangzhou Key Laboratory of Insect Development Regulation and Application Research, Institute of Insect Science and Technology, School of Life Sciences, South China Normal University, Guangzhou 510631, China; Guangzhou Key Laboratory of Insect Development Regulation and Application Research, Institute of Insect Science and Technology, School of Life Sciences, South China Normal University, Guangzhou 510631, China; Division of Plant Sciences and Technology, University of Missouri, Columbia, MO 65211, USA; Guangzhou Key Laboratory of Insect Development Regulation and Application Research, Institute of Insect Science and Technology, School of Life Sciences, South China Normal University, Guangzhou 510631, China

## Abstract

G-quadruplex structure (G4) is a type of DNA secondary structure that widely exists in the genomes of many organisms. G4s are believed to participate in multiple biological processes. Acyl-CoA binding protein (ACBP), a ubiquitously expressed and highly conserved protein in eukaryotic cells, plays important roles in lipid metabolism by transporting and protecting acyl-CoA esters. Here, we report the functional identification of a G4 in the promoter of the *ACBP* gene in silkworm and human cancer cells. We found that G4 exists as a conserved element in the promoters of *ACBP* genes in invertebrates and vertebrates. The *BmACBP* G4 bound with G4-binding protein LARK regulated *BmACBP* transcription, which was blocked by the G4 stabilizer pyridostatin (PDS) and G4 antisense oligonucleotides. PDS treatment with fifth instar silkworm larvae decreased the *BmACBP* expression and triacylglycerides (TAG) level, resulting in reductions in fat body mass, body size and weight and growth and metamorphic rates. PDS treatment and knocking out of the *HsACBP* G4 in human hepatic adenocarcinoma HepG2 cells inhibited the expression of HsACBP and decreased the TAG level and cell proliferation. Altogether, our findings suggest that G4 of the *ACBP* genes is involved in regulation of lipid metabolism processes in invertebrates and vertebrates.

## INTRODUCTION

The G-quadruplex (G4) is a noncanonical DNA secondary structure that tends to form in guanine (G)-rich sequences (1). The G4 structure is formed by two or more stacked G-tetrads, which are square coplanar arrays of four guanine bases achieved through eight Hoogsteen hydrogen bonds (2). G4 structures have been found to play important cellular roles in transcription, replication, translation and telomere protection (3–6). G4s have been regarded as new anticancer targets because they regulate the expression of some oncogenes and inhibit the catalytic function of telomerase, but increasing evidence has shown that G4s are also directly involved in normal biological processes. In the nervous system, abnormal expansion of C9orf72 GGGGCC hexanucleotide repeats causes amyotrophic lateral sclerosis, and frontotemporal dementia is related to G4s (7). G4 structures accumulate in Alzheimer's disease neurons and are associated with reduced neuronal gene expression (8). G4s participate in autophagy by affecting ATG7 expression (9). In zebrafish embryos, G4s are involved in embryonic development (10). Our previous study showed that a G4 structure activated the expression of *BmPOUM2*, which is involved in the regulation of wing development in *Bombyx mori* (11).

In the human genome, >700 000 potential G-quadruplex-forming sequences (PQSs) have been identified using high-throughput sequencing (12). In the silkworm genome, 6278 PQSs have been predicted (13). Using G4-binding compounds, such as pyridostatin (PDS) and TMPyP4, G4 structures have been indirectly verified to exist in cells *in vivo*. The expression of genes with G4 motifs can be up- or downregulated by PDS or TMPyP4 (10,14). G4 structures have also been detected *in vivo* with an engineered G4 antibody (BG4) and G4-binding proteins (11,15,16). In humans, 42.7% of genes contain one or more PQSs in their promoter regions (17), and ∼1000 endogenous G4 structures have been marked in human chromatin and nucleosome-depleted regulatory regions using G4-ChIP-seq with BG4 (18), indicating the regulatory roles of G4 in gene transcription.

Intracellular long-chain fatty acids (LCFAs) are rapidly acylated to long-chain fatty acyl-CoAs (LCFA-CoAs), which are considered classical substrates in diverse intracellular metabolic pathways for energy production (mitochondrial oxidation), formation of storage lipids (triacylglycerides [TAGs], cholesteryl esters) and phospholipid synthesis for membrane biogenesis (19). Therefore, LCFA-CoAs are at the centre stage of lipid metabolism (20). The human and mouse acyl-CoA binding proteins (ACBPs) consist of 87 amino acids that have two distinct functions, namely they bind to long-chain acyl-CoA molecules and regulate lipid metabolism (21,22), and they, as diazepam-binding inhibitors, inhibit the gamma-aminobutyric acid receptor (23). The livers of ACBP-transgenic mice exhibit increased liver LCFA-CoA pool sizes and TAG content (19). Recently, ACBP was suggested to be the ‘hunger protein’ that is overabundant in human obesity (24,25). In mice, systemic injection of ACBP protein inhibits autophagy, induces lipogenesis, reduces glycaemia, and stimulates appetite as well as weight gain (26). ACBP neutralization enhances autophagy, stimulates fatty acid oxidation, inhibits appetite, reduces weight gain in the context of high-fat diet consumption or leptin deficiency, and accelerates weight loss (27). Moreover, ACBP is associated with tumorigenesis. ACBP is highly expressed in glioblastoma multiforme and maintains a high proliferation rate of the cancer cell by mediating the availability of fatty acyl-CoA for mitochondria, promoting fatty acid oxidation (28).

In insects, ACBP has been identified in several species, such as *Bombyx mori* (29), *Manduca sexta* (30), *Helicoverpa armigera* (31) and *Rhodnius prolixus* (32). It has been reported to participate in the biosynthesis of pheromones (29,33) and ecdysteroids (31). In *B. mori*, two ACBP isoforms have been identified, pgACBP and mgACBP (29). pgACBP is predominantly expressed in the pheromone gland (29). Knockdown of pgACBP in silkworm pupae reduces the overall TAG content of cytoplasmic lipid droplets, limiting bombykol biosynthesis (34). Transcriptome analysis of *B. mori* wing discs from 5-day-old larvae, prepupae and pupae has revealed that ACBP is upregulated before pupation and downregulated after pupation (35), indicating that pgACBP not only plays a role in promoting lipid droplet accumulation in pheromone glands but also may play a regulatory role in metamorphosis. Despite the important biological role of ACBP, studies on the regulation of *ACBP* expression are scant. In rats, the relatively high basal transcription of the *ACBP* gene is modulated by members of the peroxisome proliferator-activated receptor (PPAR) and sterol-regulatory element binding protein (SREBP) subfamilies (36). In *B. mori*, a humoral factor, D-glucosyl-l-tyrosine, stimulates the transcription of *ACBP* in the pheromone gland (37). In *R. prolixus*, serotonin regulates the expression of *ACBP* (38).

In this study, the G4 structure was demonstrated, for the first time, to be involved in lipid metabolism by regulating the expression of *ACBP* in *B. mori* (*BmACBP*) and *H. sapiens* (*HsACBP*).

## MATERIALS AND METHODS

### Experimental insects

The *B. mori* strain Dazao was provided by the Research and Development Center of the Sericultural Research Institute of the Academy of Agricultural Sciences of Guangdong Province, China. Larvae were reared on fresh mulberry leaves under 14/10 h light/dark cycles at 26°C.

For the PDS feeding experiment, PDS (MCE, NJ, USA) was dissolved in water and stored at −20°C. For 4 days, newly hatched larvae were forced to feed on artificial fodder that was supplemented with 20 μl of PDS solution (15 mM) per day to ensure that the larvae ate all the diet. The same amount of diet with water was supplied for the control group. Thirty larvae were used for each experimental group.

For PDS injection, 2 μl of 15 mM PDS was injected into 2-day-old fourth-instar larvae (L4D2). The same volume of deionized distil water was injected in the controls. Thirty larvae were used for each experimental group. The fat body and epidermis were isolated at 24, 48 and 72 h post-injection.

In addition, 2, 4 or 6 μl of 30 mM PDS was injected into the lateral side of thorax between the second and third segments of the last fifth-instar larvae at the early spinning stage. Six microlitres of deionized distil water was injected into the controls. The total numbers of injected larvae were 46, 76 and 60 for the PDS injection volumes of 2, 4 and 6 μl, respectively. The total number of injected larvae in the control group was 85. The fat body and wing discs were isolated at 12, 24 and 36 h post-injection. All experiments were performed with three biological replicates.

### Cell culture

The *B. mori* cell line DZNU-Bm-12 (*Bm12*), originally developed from ovarian tissues (39), was maintained at 28°C in Grace medium (Gibco, New York, USA) supplemented with 10% foetal bovine serum (FBS, Thermo Scientific, MA, USA). The Chinese hamster ovary (CHO) cell line was maintained at 37°C in F-12K Nutrient Mixture medium (Gibco, New York, USA) supplemented with 10% FBS. HepG2 cells were purchased from ATCC (American Type Culture Collection, VA, USA) and grown in DMEM (Thermo Scientific, MA, USA) supplemented with 10% FBS and 100 U/ml penicillin–streptomycin (Thermo Scientific, MA, USA) at 37°C. All cell stocks were regularly tested for mycoplasma contamination. For PDS treatments, different concentrations of PDS were added to 1 ml of culture medium in each of the cell culture wells. The cells were cultured for 48 h and then collected for RNA isolation and quantitative real-time polymerase chain reaction (qRT–PCR) assays.

### Circular dichroism (CD) assay

CD analyses were conducted by using a J-815 CD spectrometer (Jasco International, Tokyo, Japan). The DNA oligonucleotide sequences were diluted to 5 μM final concentration using 50 mM Tris–HCl (pH 7.5). To fold the single-stranded DNA (ssDNA) into the G4 structure, the oligonucleotide was heated at 95°C for 10 min in 50 mM Tris-buffer (pH 7.5) with or without 100 mM KCl and then slowly cooled to room temperature over 4 h to allow the G4 structure to form. All spectra were obtained at wavelengths ranging from 220 to 350 nm with a 1 nm step width and a 1 s response time. The CD spectra data represent three averaged scans from the same sample and were baseline-corrected for the signal contributions derived from the buffer used to dissolve the DNA oligonucleotides.

### Determination of TAG amounts

The third thorax fat body or thoracic section without a coating of intestinal matter was added to 200 μl of Glycerol Lysis Solution (Promega, WI, USA, J3160), homogenized for 30 s, and centrifuged for 1 min at 12 000 × g at 4°C. The supernatant was collected and used as a test sample. TAG content was measured using a Triglyceride-Glo Assay kit (Promega, WI, USA, J3160). The TAG concentration is expressed in micromoles.

### Expression and purification of recombinant BmLARK protein

Protein expression was performed using the previously reported plasmid BmLARK-pGEX-6P-1, which contains a GST marker gene (11). The recombinant BmLARK protein was expressed in *Escherichia coli* cells (BL21) cultured in Luria Bertani medium containing 100 μg/ml ampicillin at 37°C with a 12 h induction by isopropyl-β-D-thiogalactoside (final concentration, 0.1 mM). Cells were collected by centrifugation at 10 000 × g for 5 min and resuspended in PBS (136 mM NaCl, 1.1 mM K_2_HPO_4_, 2.7 mM KCl and 8.0 mM Na_2_HPO_4_, pH 7.4). The suspension was lysed by sonication on ice and then centrifuged at 10 000 × g at 4°C for 5 min. The recombinant GST-LARK protein in the supernatant was purified using BeyoGold GST-tag Purification Resin (Beyotime, Beijing, China). The purified protein was then dialyzed using a 25 kDa molecular weight cut-off membrane (Millipore, MA, USA) against 20 mM Tris–HCl buffer (pH 7.5) at 4°C overnight. The protein concentration was measured using a BCA Protein Assay Reagent Kit (Thermo Scientific, MA, USA). The purified protein was examined with sodium dodecyl sulfate–polyacrylamide gel electrophoresis (SDS-PAGE).

### Electrophoretic mobility shift assay (EMSA)

EMSA was performed according to the instructions of a Light Shift Chemiluminescent EMSA Kit (Thermo Scientific, MA, USA). The wild-type and mutant oligonucleotides labelled with 6-FAM at the 5′ terminus (Invitrogen, CA, USA) were heated at 95°C for 10 min in 50 mM Tris-buffer (pH 7.5) with or without KCl and slowly cooled to room temperature over 4 h. The concentration of the DNA oligonucleotides was 10 μM.

The binding reactions were conducted at room temperature in a 20 μl reaction system containing 1 × binding buffer (10 mM Tris, 50 mM KCl, 1 mM DTT, pH 7.5), 2 μg of purified recombinant LARK protein or 1 μg of BG4 (Ab00174-10.6, Absolute Antibody, UK) and 6-FAM-labeled probes with a final concentration of 2 μM for 20 min. For the competition assay, cold probes (the same DNA sequences, but unlabelled) were added to the binding reaction. For PDS treatment, different final concentrations (1, 2 and 3 μM) of PDS were incubated with 2 μM 6-FAM-labelled probe for 30 min before or after the binding reaction. Then, the samples were separated on a 4% polyacrylamide gel on ice at 100 volts for 1.5 h. The signal was captured using Bio-Rad ChemiDoc Touch imaging system. The oligonucleotide probes used in this study are shown in Supplementary Table S1.

### Immunostaining

Cells grown on glass coverslips were fixed in 4% paraformaldehyde for 10 min, permeabilized with 0.5% Triton X-100 at room temperature, and then blocked in a blocking solution (2% bovine serum and 5% goat serum in PBS) for 1 h at 37°C. For G4 staining, the cells were incubated with BmLARK-GST (10 μg/ml) in a 1:10 diluted blocking solution for 2 h at room temperature. The cells were incubated with a primary anti-GST antibody (Abcam, Cambridge, UK) at a 1:500 dilution in 1:10-diluted blocking solution overnight at 4°C. On the following day, the cells were incubated with Alexa 488-conjugated anti-mouse secondary antibodies (Invitrogen, CA, USA) at a 1:1000 dilution in 1:10-diluted blocking solution for 1 h at 37°C. The cells were washed with PBS three times before each procedure. Then, the coverslips were mounted with Prolong Gold/DAPI (Invitrogen, CA, USA), and confocal images were obtained under an Olympus Fluoview FV1000 confocal microscope. For Ki-67 and ACBP staining, the cells were incubated with an anti-Ki-67 antibody (Proteintech Group, IL, USA) and an anti-ACBP antibody (Santa Cruz Biotechnology, TX, USA) at a 1:400 dilution and then with Alexa 488-conjugated anti-mouse (Invitrogen, CA, USA) and Alexa 594-conjugated anti-rabbit secondary antibodies at a 1:1000 dilution.

### Cellular lipid staining

5 × 10^5^ wildtype or G4-mutant human hepatic adenocarcinoma HepG2 cells were inoculated in 12-well culture plates and cultured for 24 h and then stained with Nile Red reagent (MCE, NJ, USA) to visualize the lipids with an Olympus Fluoview FV1000 confocal microscope.

### Chromatin immunoprecipitation (ChIP) assay

A ChIP assay was carried out as previously described (11). Briefly, Bm12 cells were transfected with BmLARK-3 × FLAG-EGFP (with a terminator codon added before the EGFP coding sequence) or EGFP (control) plasmids for 48 h and then crosslinked with 1% formaldehyde for 10 min at room temperature. Glycine was used to terminate the fixation. Digested chromatin was obtained by sonication. The immunoprecipitation (IP) experiment was performed with a Pierce Magnetic ChIP Kit (Thermo Scientific, Massachusetts, USA) according to the manufacturer's instructions. Ten micrograms of either rabbit anti-FLAG antibody (#14793, Cell Signaling Technology, MA, USA) or normal rabbit IgG (as a control) (Thermo Fisher Scientific, MA, USA) were used for the IP reactions. The immunoprecipitated genomic DNA fragments were amplified using qRT–PCR with primers (Supplementary Table S1). The specificity of the primers was examined using Primer-BLAST (http://www.ncbi.nlm.nih.gov/tools/primer-blast/). The enrichment of the promoter sequences in the immunoprecipitated DNA samples was normalized to the DNA present in the 10% input material as analysed using the 2^−ΔΔCt^ method (40). The polymerase chain reaction (PCR) products of the enriched fragments were sequenced by Tsing Ke Biotechnology (Guangzhou, China).

### Cleavage under targets and tagmentation (CUT&Tag)

CUT&Tag was performed essentially as described by Kaiwei Liang (41), with minor modifications according the Hyperactive Universal CUT&Tag Assay Kit protocol (Vazyme, Nanjing, China). Bm12 cells and HepG2 cells (1 × 10^6^) were used for the CUT&Tag experiment, and cell counting was performed by using a Cellometer Mini (Nexcelom Bioscience, Boston, MA, USA). pA-Tn5 transposase was used to cut the genome and add a special adaptor sequence to build a library. The enrichment of target sites in the library was detected using qRT–PCR. The G4 antibody BG4 was purchased from Sigma (MABE917, Shanghai, China).

The CUT&Tag reads were aligned to the human genome (UCSC hg38) with Bowtie2. The aligned reads were removed with Picard MarkDuplicates, and the deduplicated BAM files were normalized to the total aligned reads (reads per million, RPM) with the bamCoverage command from deepTools 3.3.0.

### Transient cell transfection

Transient cell transfection and cotransfection were performed using FuGENE HD Transfection Reagent (Promega, Madison, WI, USA) as suggested by the manufacturer. For BmLARK overexpression, 2 μg of BmLARK-3 × FLAG-EGFP plasmids were transfected into Bm12 cells. The cells were collected 48 h after transfection. Western blotting and qRT–PCR were performed to detect the efficiency of protein overexpression. For the antisense oligonucleotide (ASO) disturbance assays, 2 μl of ASOs (100 mM) were transfected into Bm12 cells, which were then collected 48 h after transfection. qRT-PCR was performed to detect the effects of the ASOs on the mRNA levels of *BmACBP*. A random sequence that did not match the ASO sequence was designed as a control. The oligonucleotides used in the ASO disturbance assays are shown in Supplementary Table S1.

### Polymerase stop assay (PSA)

A PSA was conducted with the wild type or mutant ACBP G4 sequence as a template. The template DNA in 50 mM Tris-HCl (pH 7.4) with 100 mM KCl was heated at 95°C for 10 min and then slowly cooled to room temperature to allow the G4 structure to form. The annealed template was incubated with different amounts of PDS for 30 min at room temperature. PCR was performed with the 5′ FAM-labeled 5′-TACTCGACAGTAACCTACCCCC-3′ primer. The mixtures for PCR were incubated at 94°C for 3 min and then subjected to 30 cycles of 94°C for 30 s, 58°C for 30 s and 72°C for 30 s. The PCR products were detected by electrophoresis in a 15% denaturing polyacrylamide gel. The concentrations of the template and the primer in the mixtures are 5 and 2.5 μM.

### Cas9-mediated genome editing

An online tool (http://crispor.tefor.net/crispor.py) was used to design the small-guide RNA (sgRNA) sequence and predict possible off-target sites.

Our approach for producing mutant animals followed the Cas9-mediated genome editing protocol reported by Zhang *et al.* (42). In general, sgRNAs were designed based on the N_20_NGG rule. An sgRNA template was produced by PCR amplification with a forward primer encoding a T7 polymerase-binding site and an sgRNA target site and a reverse primer encoding the remainder of the sgRNA sequence (Supplementary Table S1). sgRNAs were transcribed *in vitro* using T7 RiboMAX Express RNAi System Kits (Promega, Madison, WI, USA) and purified by phenol/chloroform extraction followed by isopropanol precipitation. Two micrograms of TrueCut™ Cas9 Protein v2 (Thermo Fisher Scientific, MA, USA) and 15 μg of sgRNA were mixed in a 5 μl volume prior to injection. *B. mori* eggs were collected within 3 h after oviposition and lined up on a piece of adhesive tape on a microscope slide. Microinjection of *B. mori* embryos was conducted using a pulled borosilicate glass needle (Sutter Instrument, ID: 0.5 mm, Novato, CA, USA) at an injection pressure of 20 psi. The treated embryos were then incubated in a chamber at 28°C and 70% humidity for the remainder of development. To confirm the mutation positions, genomic DNA was extracted from single caterpillars or last-instar larvae using digestion buffer (1 M Tris-Cl, 0.5 M EDTA, 5 M NaCl, 10% SDS, 60 mg/ml proteinase K) and a PCR product purification kit (Magen, Guangzhou, China). The fragments flanking the Cas9 target regions were amplified by PCR using primers flanking the deletion (Supplementary Table S1). A total of seven off-target sites were predicted: two in intronic regions and five in intergenic regions (Supplementary Table S2). We detected these seven possible off-target sites by sequencing, and the results showed that real off-target effects did not occur (Supplementary Figure S1).

The sgRNA targeting the G4 site (Supplementary Table S1) of *hACBP* was cloned into the BbsI site of the pSpCas9(BB)-2A-Puro (PX459) V2.0 vector according to the methods of Ran et al. (43). The vectors were transfected into HepG2 cells with Lipofectamine™ 3000 (Thermo Scientific, MA, USA) according to the product protocol. At 48 h post-transfection, the medium was replaced with fresh medium containing puromycin (0.5 ug/ml), and the cells were maintained for 10 days with medium changes every 2 or 3 days. On the 10th day of puromycin selection, the cells were separated into single cells by the limiting dilution method in 96-well plates.

### RNA isolation, reverse transcription, and quantitative real time PCR (qRT–PCR)

Total RNA was extracted from the frozen tissue samples or cell lines using Eastep Super Total RNA Extraction Kit (Proemga, WI, USA). The residual genomic DNA was removed by DNase treatment. The concentration of RNA was determined by NanoDrop 2000 spectrophotometer (Thermo Fisher Scientific, MA, USA). The cDNA was synthesized with GoScript Reverse Transcription Mix (Proemga, WI, USA). qRT-PCR was performed with SYBR Premix Ex Taq II Green Real-time PCR Master Mix (TaKaRa, Dalian, China) following previously reported procedures (44). The relative expression levels of the genes were normalized to the expression of *Bombyx mori* ribosomal protein 49 (BmRP49) or *Homo sapiens* lyceraldehyde-3-phosphate dehydrogenase (HsGAPDH) and analysed using the 2^−ΔΔCt^ method (40). The primer sequences are listed in Supplementary Table S1. Three biological and technical repeats were performed for each treatment. The data are presented as the mean ± SEM. Significance was analysed using Student's *t* test with GraphPad Prism 6 (GraphPad Software Inc.).

### Western blot analysis

Protein was extracted from cells or silkworm fat bodies using 1 × RIPA buffer supplemented with 1 × Protease Inhibitor (Bestbio, Shanghai, China). The protein samples were added to 6 × Protein Loading Buffer (Transgen, Beijing, China) and then immediately denatured at 80°C for 10 min. The samples were analysed using 12% SDS-polyacrylamide gels. The proteins were transferred to PVDF membranes (MILLIPORE, Billerica, MA, USA). Immunoblotting was carried out according to standard procedures using Super ECL Detection Reagent (YEASEN, Shanghai, China). The membranes were hybridized with monoclonal anti-ACBP (1:1000 dilution; sc-376853, Santa Cruz), anti-β-Tubulin (1:2000 dilution; TB002-R or TB002-M, Guangzhou Dingguo Biology, China), and anti-LARK (1:1000 dilution) antibodies.

### Bioinformatics analysis

Gene ontology (GO) enrichment analysis was conducted with the genes containing PQSs at 2 kb upstream of the transcription start site (TSS) using the GO enrichment analysis program on the OmicShare website (https://www.omicshare.com/tools/Home/Soft/gogsea). The GO background file is listed in Dataset File 1.

### Statistical analysis

Statistical analysis was conducted using GraphPad Prism software (GraphPad Software Inc.). Three biological and technical repeats were performed for each condition. The results of qRT–PCR assays are presented as the mean ± SEM. Comparisons between two groups for a single parameter were conducted using Student's *t* tests (one unpaired *t* test per row). The criteria for statistical significance are indicated by asterisks (*P < 0.05, **P < 0.01 and ***P < 0.001) in the figures.

## RESULTS

### The promoters of the *ACBP* genes in multiple species contain PQSs

In our previous study of *B. mori*, we identified 332 genes that contain PQSs in their promoters (45). The GO analysis results showed that the genes containing PQSs were significantly enriched in the lipid-related pathways (Supplementary Figure S2). In particular, the *ACBP* gene (*BmACBP*) attracted our attention.

**Figure 1.**
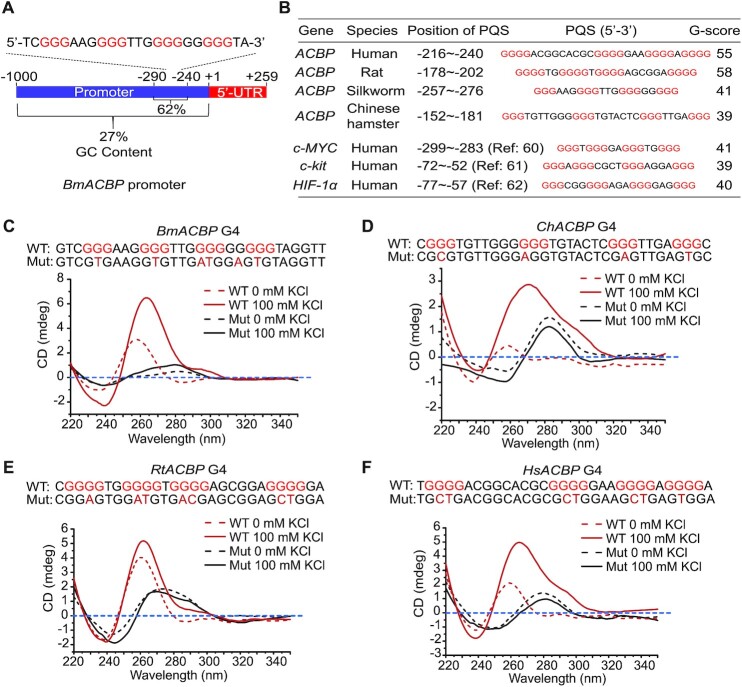
Identification of the G4 structure in the *ACBP* promoter. (**A**) Analysis of the *ACBP* promoter sequences. Sequences 1 kb upstream of the TSS were analysed. (**B**) Comparison of the PQSs of the *ACBP* promoters in different species and some reported G4-containing sequences (60–62) predicted by QGRS Mapper. (**C–F**) CD analysis of the forward ssDNA of the –257/–276 nt region of the *ACBP* promoter in the absence or presence of 100 mM KCl in different species. The mutated nucleotides are shown in the mutant sequence with red font. WT: wild-type strand; Mut: mutated strand; Bm: *Bombyx mori*; Ch: Chinese hamster; Hs: *Homo sapiens*; Rt: rat.

Sequence analysis and PQS prediction showed that the GC content in the promoter region from –290 to –240 of *BmACBP* was 62% (Figure 1A) and that the –257∼–276 region could form a PQS. G4 prediction analysis of the promoters of the *BmACBP* homologues in humans (GenBank accession number: NM_020548.9), rats (GenBank accession number: NM_031853.4) and Chinese hamsters (GenBank accession number: XM_027391815.1) also showed similar PQSs with high G-scores (Figure 1B). We aligned the upstream 1 kb regulatory region prior to the transcriptional start site (TSS) and the G4 sequences of ACBPs in 12 species and found that the G-tracts of the G4 sequences are evolutionarily conserved, while the loops are not exactly the same (Supplementary Figure S3).

To confirm the formation of the predicted G4 structures, CD analysis was performed after a process of denaturation and renaturation in the presence of K^+^, which allowed the G4 structure to form. The CD results showed that the G4 sequences in the promoters of human, silkworm, rat and Chinese hamster *ACBPs* had a maximum absorption peak at 265 or 270 nm and a minimum absorption peak at the 240 nm, exhibiting specific and typical features of parallel G4 structures (Figure 1C-F). These results suggest that the predicted G4 sequences in the promoter regions of the silkworm, human, rat and Chinese hamster *ACBP* homologues can form the G4 structure *in vitro*.

### The PQS of *BmACBP* folds into the G4 structure *in vitro* and *in vivo*

**Figure 2.**
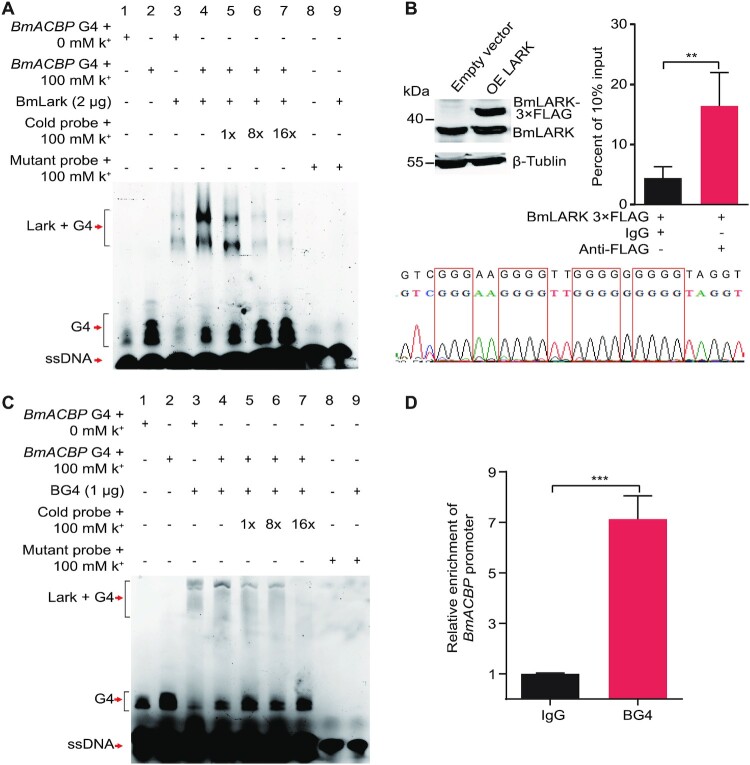
Binding of *BmACBP* G4 with G4-binding proteins. (**A, C**) EMSA of the interaction between the G4 structure and the G4-binding protein BmLARK or the G4-specific antibody BG4. The positions of the labelled G4 probe, labelled ssDNA probe and labelled bound G4 and BmLARK are shown by arrows. (**B, D)** ChIP analysis of the binding between BmLARK and BG4 with G4 structures in Bm12 cells. The enrichment of the promoter sequence in immunoprecipitated DNA samples was normalized to the DNA present in the 10% input material. The data are the mean ± SEM (*n* = 3). **P < 0.01, ***P < 0.001 (Student's *t* test).

To further confirm the G4 structure of *BmACBP*, EMSA was performed using the previously identified G4-specific binding protein BmLARK (11) and a G4-specific antibody BG4 (15). The results showed that in the presence of K^+^, the *BmACBP* PQS could form a G4 structure (Figure 2A and C, lanes 1 and 2). BmLARK and BG4 specifically bound to the G4 structure (Figure 2A and C, lanes 3 and 4). The binding intensity was reduced when increased concentrations of cold probes were added (Figure 2A and C, lanes 5–7). BmLARK and BG4 did not bind to the mutant G4 sequence, which could not form the G4 structure (Figure 2A and C, lanes 8 and 9). These results indicated that the predicted G4 sequence in the *BmACBP* promoter could form a G4 structure *in vitro* and be recognized by G4-binding proteins.

To further demonstrate the *in vivo* formation of the G4 structure in the *BmACBP* promoter, a ChIP experiment was conducted using a commercial FLAG antibody in Bm12 cells in which the BmLARK protein with a 3 × FLAG label was overexpressed. The anti-FLAG antibody, but not IgG, precipitated and significantly enriched the G4 structure-containing DNA of the *BmACBP* promoter from the cells (Figure 2B), demonstrating the presence of the G4 structure in the *BmACBP* promoter. Additionally, a CUT&Tag experiment was conducted using the G4 antibody BG4. The results showed that compared to the control IgG, BG4 could significantly enrich the G4-containing DNA of the *BmACBP* promoter (Figure 2D). Taken together, all the results of the *in vitro* and *in vivo* experiments demonstrated the presence of the G4 structure in the *BmACBP* promoter.

### The G4 structure promotes the transcription of *BmACBP*

**Figure 3.**
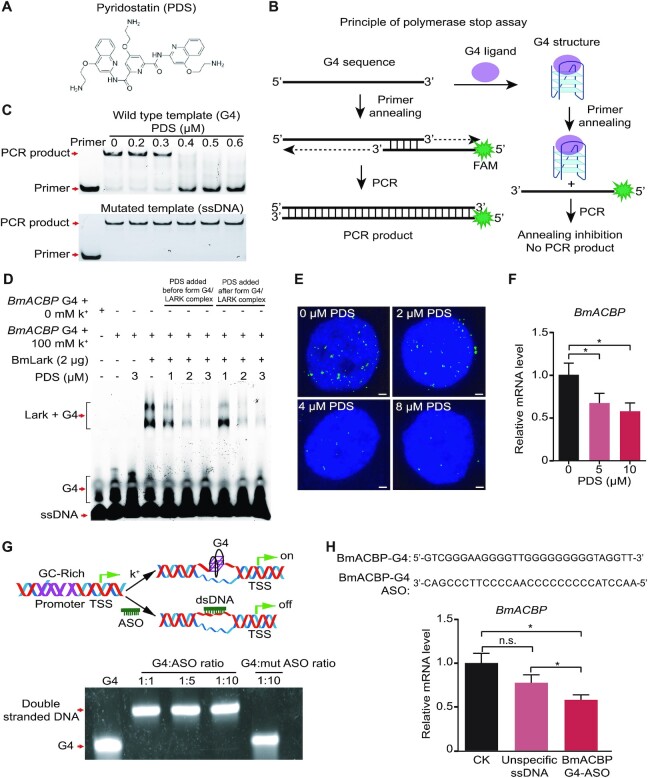
Transcriptional regulation of *BmACBP* by the G4 structure. **(A**) Structure of the compound PDS. (**B**) Principle of polymerase stop assay. The G4 sequence is amplified with a complementary FAM labelled primer. The PCR product can be obtained without G4 ligand. In the presence of G4 ligand that stabilizes G4 sequences into G4 structures, therefore, the primer annealing and polymerase extension are inhibited. There are no PCR product to be obtained. (**C)** PCR in the PSA with different concentrations of PDS. (**D**) EMSA of the inhibitory effect of PDS on the binding of BmLARK to the G4 structure. (**E**) Immunofluorescence showing the inhibition of BmLARK binding with G4 structures by PDS in Bm12 cells. All scale bars, 1 μm. (**F**) The mRNA level of *BmACBP* in Bm12 cells after treatment with PDS. (**G**) Pattern of disruption of the G4 structure by ASOs. The G4 structure was disrupted by ASOs *in vitro*. (**H**) The mRNA level of *BmACBP* in Bm12 cells after treatment with ASOs. The data are the mean ± SEM (*n* = 3). *P < 0.05, n.s. (nonsignificant) = *P* > 0.05 (Student's *t* test).

To investigate the regulatory function of the G4 structure in *BmACBP* transcription, the effects of PDS (Figure 3A) and ASO treatments on *BmACBP* transcription were studied.

PDS is a reagent that can specifically bind with the DNA G4 structure (46,47). In this study, PSA was first performed to verify the PDS stabilization of the *BmACBP* G4 structure (Figure 3B). The intensity of the target PCR product band decreased when the concentration of PDS was increased in the PCR system, and PCR amplification was completely suppressed when the PDS concentration was 0.4 μM (Figure 3C, top panel), and the PDS effect disappeared when the mutated PCR template, which cannot form a G4 structure, was used (Figure 3C, bottom panel). Since PDS can specifically bind with and stabilize the G4 structure, the stabilized G4 structure blocks gene transcription, resulting in fewer or no product in PCR reaction. The results indicated that the G4 structure was formed and bound by PDS.

In addition, the EMSA experiment showed that the binding of BmLARK to the G4 structure was inhibited by PDS (Figure 3D). Regardless of whether it was added before or after the binding of BmLARK with G4, PDS inhibited the development of the binding bands. To further confirm the inhibitory effect of PDS on the BmLARK binding to G4 structures in cells, recombinant BmLARK-GST protein and an anti-GST antibody were used in an immunohistochemistry assay. The binding of BmLARK to the G4 structures in the cell nuclei was visualized by fluorescence signals generated using a fluorescence-labelled secondary antibody. With no PDS treatment, punctate nuclear staining was detected in Bm12 cells (Figure 3E). When the cells were treated with PDS, an obvious decrease in nuclear staining was observed, and the inhibitory effect increased with increasing PDS concentrations (Figure 3E), implying that PDS blocked the LARK protein binding to G4 structures, probably by occupying the binding site in the G4 structure when the compound stabilizes the structure. Furthermore, Bm12 cells were treated with PDS to test its effect on *BmACBP* transcription. The results revealed that *BmACBP* transcription was significantly inhibited by PDS compared to the control (Figure 3F). These results indicated that the occupation of the G4 structure by PDS suppressed the interaction of the protein and G4 structure, leading to suppressed *BmACBP* transcription.

An ASO experiment was performed to specifically inhibit the formation of the G4 structure in the *BmACBP* promoter. If complementary oligos are present at high levels, DNA tends to form a double-stranded structure rather than a G4 structure (Figure 3G). G4 structure formation was inhibited when oligonucleotides complementary to the G4 sequence were added to the reaction system, while when the mutant oligonucleotides were added, G4 structure could form (Figure 3G). DNA oligos complementary to the G4 sequence were transfected into Bm12 cells, and *BmACBP* expression was detected (Figure 3H). The results showed that *BmACBP* expression was inhibited when the formation of the G4 structure was suppressed by G4 ASOs.

All of the above results of PDS and ASO treatments indicated that the expression of *BmACBP* was downregulated when the G4 structure was either competitively prevented from binding with BmLARK by PDS or disrupted by ASOs.

### PDS reduces lipid accumulation by inhibiting the expression of BmACBP in *Bombyx mori*

**Figure 4.**
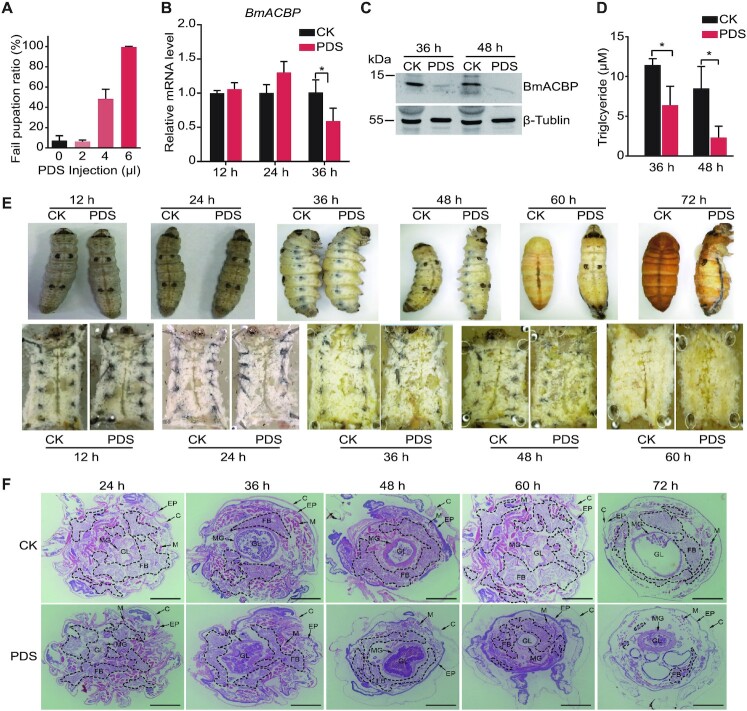
Effect of PDS on the development of prepupal *B. mori*. **(A**) Ratio of larvae that unsuccessfully pupated after PDS injection. (**B**) mRNA expression of *BmACBP* after PDS injection in *B. mori*. (**C**) Western blot analysis of BmACBP protein levels after PDS injection in *B. mori*. (**D**) Values for fat body TAG accumulation. (**E**) Morphological and anatomical comparison between the experimental group and the control group. The white tissues in the anatomical images (bottom panel) are fat bodies. (**F**) Haematoxylin and eosin (HE) staining of cross-sections of *B. mori*. PDS was injected into the lateral side of thorax between the second and third segments of the last fifth-instar larvae at the early spinning stage, and the cross-sections were obtained at corresponding time points. Fat bodies are surrounded by black dotted lines. FB: fat body; C: cuticle; EP: epidermis; M: muscle; MG: midgut; GL: gut lumen. The data are the mean ± SEM (*n* = 3). *P < 0.05 (Student's *t* test). All scale bars, 1 mm.

To further determine the function of the BmACBP G4, 5^th^-instar larvae were injected with 2, 4 or 6 μl of 15 mM PDS. The results showed that the larvae treated with 2 μl of PDS were able to complete the transition from larvae to pupae, while 50% and 100% of larvae treated with 4 and 6 μl of PDS failed to complete the transition from larvae to pupae (Figure 4A and E). We further chose those larvae treated with 6 μl of PDS and tested the expression of *BmACBP* at the mRNA and protein levels. The results showed that *BmACBP* mRNA levels significantly decreased after PDS treatment for 36 h (Figure 4B), while BmACBP protein levels significantly decreased after PDS treatment for 36 and 48 h (Figure 4C). Since, the ACBP gene was reported to involve TAG storage (19). We assessed TAG levels and found that they significantly decreased after PDS treatment (Figure 4D). In addition, dissection and tissue slice observation revealed that fat body dissociation was aggravated. In the control larvae, a large amount of fat body was found, but in the PDS-injected larvae, the amount of fat body was decreased or even absent (Figure 4E and F).

These results indicate that the promoter of BmACBP can form a G4 structure and that compounds targeting the G4 structure can regulate the expression of *BmACBP*, resulting in loss of fat body.

### Regulation of *HsACBP* expression by targeting of G4 in human cells

**Figure 5.**
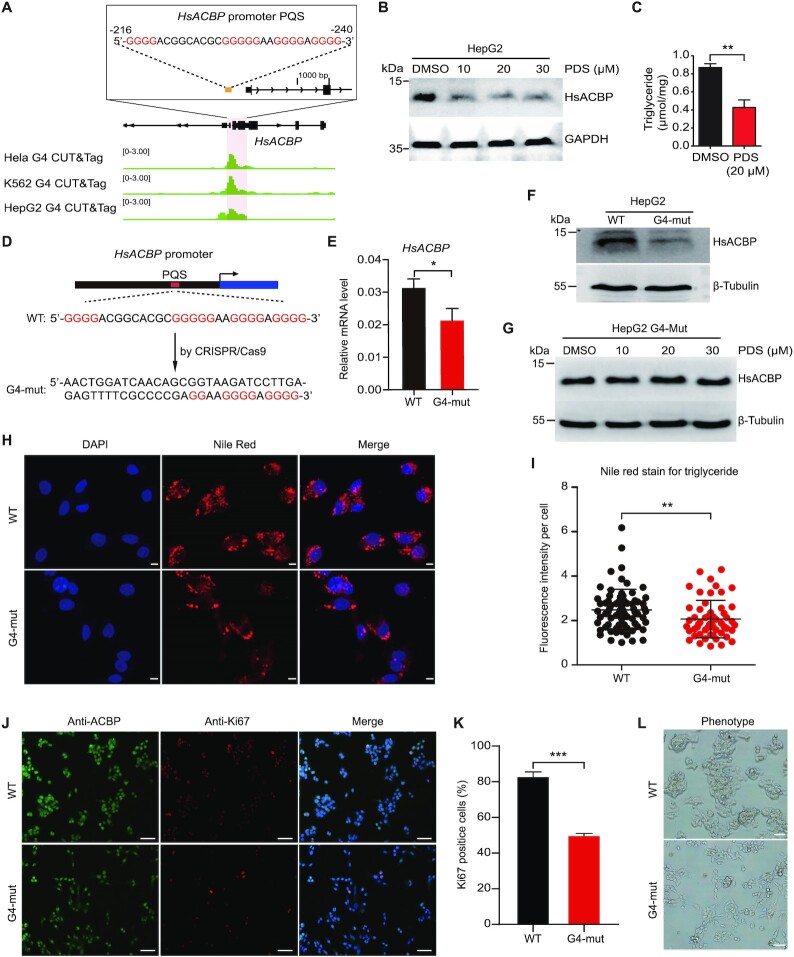
Regulation of the expression of *HsACBP* by targeting of G4 in human HepG2 cells. (**A**) G4 CUT&Tag signals at the *HsACBP* promoter loci. G4 CUT&Tag data for HeLa and K562 cells were obtained from the NCBI Gene Expression Omnibus database (GSE178668) (41). G4 CUT&Tag data for HepG2 cells were generated in this study. (**B**) Western blot analysis of HsACBP after PDS treatment. (**C**) Detection of TAG content after treatment of HepG2 cells with 20 μM PDS. (**D**) The *HsACBP* promoter PQS was mutated by CRISPR–Cas9. (**E, F**) mRNA and protein levels of *HsACBP* in G4-wild-type and G4-mutant cells. (**G**) Western blot analysis of HsACBP after PDS treatment in G4-mut HepG2 cells. (**H, I**) Representative images and quantification of Nile red staining for TAG in G4-wild-type and G4-mutant cells. All scale bars, 5 μm. (**J**) Representative immunofluorescence staining of G4-wild-type and G4-mutant cells for ACBP and the proliferation marker Ki67. All scale bars, 50 μm. (**K**) Quantification of the Ki67 index. The data in C, E and J are the mean ± SEM (*n* = 3); the data in H are the mean ± SD (n > 50). *P < 0.05, **P < 0.01, ***P < 0.001 (Student's *t* test). (**L**) Phenotypes of G4-wild-type and G4-mutant cells. The proliferation of G4-mutant cells was significantly inhibited. All scale bars, 50 μm.

*ACBP* is a highly conserved gene, and PQSs are located at similar positions in *ACBP* promoter regions in many species (Figure 1). We speculated that the function of *ACBP* G4s in regulating transcription is also conserved. Therefore, we further explored the effects of human *HsACBP* G4 on gene transcription and lipid metabolism in human hepatic adenocarcinoma HepG2 cells. To investigate whether the *HsACBP* promoter region forms a G4 structure in cells, we analysed BG4 CUT&Tag data of HeLa and K562 cells obtained from the NCBI Gene Expression Omnibus database (GSE178668) (41). We found that the G4 was significantly enriched in the promoter of human *ACBP* (Figure 5A). We performed CUT&Tag experiments with HepG2 cells and found that the results were consistent with those in HeLa and K562 cells and the G4 was also significantly enriched in the *HsACBP* promoter (Figure 5A). Then, we treated HepG2 cells with PDS, and similar to the findings for silkworms, the expression levels of *HsACBP* were decreased significantly by the PDS treatment (Figure 5B). The TAG levels were also significantly reduced (Figure 5C). These results suggest that a G4 structure can fold in the promoter of *HsACBP* in the cellular context and regulate the transcription of *HsACBP*.

Although PDS is a highly specific drug for G4 structures, changes in a particular gene in PDS treatment experiments cannot be directly explained by G4 structures. To further demonstrate the function of the G4 in the regulation of *HsACBP*, we directly mutated the *HsACBP* G4 sequence by CRISPR–Cas9. One mutant cell line was obtained after monoclonal screening and confirmed by DNA sequencing. In the mutant cell line, a 16 bp sequence of *HsACBP* G4 was replaced with a 44 bp sequence (Figure 5D), which should result in failure of the G4 formation. qRT-PCR and Western blot analyses proved that the expression of *HsACBP* at both the mRNA and protein levels was significantly decreased in the mutant cells compared to the wild-type cells (Figure 5E and F). We then tested the effect of PDS on the ACBP expression after G4 mutation. The result showed that when the *ACBP* promoter G4 was mutated, PDS no longer inhibited the expression of this gene (Figure 5G), indicating that the effect of PDS was through the G4 structure. We then tested the TAG content by Nile red staining in mutant and wild-type cells and found that it was significantly decreased in the G4-mutant cells (Figure 5H and I). Reductions in ACBP have been shown to decrease glioblastoma proliferation (27,28). Consistent with previous studies (27,28), we performed Ki67 staining, which is an indicator of cell proliferation and found that Ki67 levels (Figure 5J and K) and cell proliferation (Figure 5L) were decreased in G4-mutant cells, indicating that cell proliferation was suppressed by the *HsACBP* G4 knockout.

## DISCUSSION

Many studies have shown that PQSs are widely distributed in the genomes of many species (12,17,48). An increasing number of analyses have revealed that these PQSs are not randomly distributed; instead, they are enriched in the region proximal to the TSS of genes. The formation of G4s in the promoter region is coupled with negative chromosomal superhelicity in the wake of the transcription bubble (49) and is linked to regulatory, nucleosome-depleted chromatin and elevated transcription (18), highlighting the possible function of G4s in gene transcription. In humans, >40% of genes, including some of oncogenes, contain one or more G4 motifs in their promoters (17). In addition to multiple oncogenes, such as *c-myc*, *Th* and *c-kit* (3,17,50), G4 structures have been demonstrated to participate in the transcriptional regulation of development-related genes. In zebrafish embryos, G4 structures regulate the transcription of the development-related genes *col2a1*, *fzd5* and *nog3* (10). We have demonstrated previously that G4 structure in the promoter of the transcription factor gene *BmPOUM2* participates in the transcription of the gene in collaboration with its binding protein BmLARK, a vital transcription factor (11). In this study, GO enrichment analysis of genes containing PQSs in their promoter regions revealed that these genes were enriched in different metabolic and developmental pathways, such as fatty acyl-CoA binding, fat response, steroid hormone mediation, the steroid hormone receptor pathway, gene transcriptional regulation and DNA binding, suggesting that the DNA G4 structure is probably involved in the transcriptional regulation of the genes responsible for these metabolic processes (Supplementary Figure S2).

PDS is a G4-targeting small molecule and has been demonstrated to specifically bind and stabilize G4 structures (14,48,51,52). In a fluorescence resonance energy transfer (FRET) melting experiment using a human telomere G-tetrad-forming sequence and double-stranded DNA, PDS exerted the strongest stabilization effect on the G4 structure at a concentration of 1 μM without affecting double-stranded DNA (46). PDS promotes growth arrest in human cancer cells by inducing replication- and transcription-dependent DNA damage. In cancer cell lines (HeLa, U2OS and HT1080) and normal cell lines (WI-38), PDS significantly inhibits cell growth after 72 h, with IC_50_ values ranging from 0.89 to 10 μM. The selectivity of PDS in HT1080 cells is 18 times higher than that in normal cells (53). Recently, the single-molecule visualization PDS derivative SiR-PyPDS (20 nM) was used to detect G4s in living cells, and chemical inhibition of transcription led to abrogation of detectable G4s (47). PDS treatment in human cancer cells promotes telomere dysfunction and long-term growth inhibition (53). In addition, stabilization of the G4 structure by PDS inhibits the transcription of *Bcl-2* (54). In previous studies, PDS has been shown to regulate gene transcription by blocking protein binding to the G4 structure (55,56). In this study, we first demonstrated that PDS stabilized the ACBP G4 (Figure 3B) and blocked the binding of the LARK protein to the G4 structure (Figure 3C and D). Then, PDS feeding and injection treatments were conducted with larvae at different developmental stages to investigate the effects of PDS through the G4 structures on the larval development of *B. mori*. Compared to the control larvae, PDS-treated larvae showed reduced feeding, reduced fat body mass, smaller body sizes, lower body weights, slower growth, and failure to moult (Supplementary Figures 4 and 5 and Figure 4). These phenotypes suggested that when the G4 structures in the cells were stabilized by PDS, the growth and development of *B. mori* larvae were substantially impacted. This is probably because that PDS occupies the site that G4 binding protein(s) binds to in the G4 structure, while the compound stabilizes the structure. Phenotypic and GO enrichment analyses revealed that the fat body and fat metabolism pathways were significantly affected by the PDS-mediated stabilization of G4 structures. Among other G4-containing genes, *BmACBP* was identified to be involved in lipid metabolism. This gene contains a G4 structure in its promoter, and its transcription level was significantly reduced upon PDS treatment, suggesting that it plays critical roles in the regulation of *B. mori* growth and development through its G4 structure.

**Figure 6.**
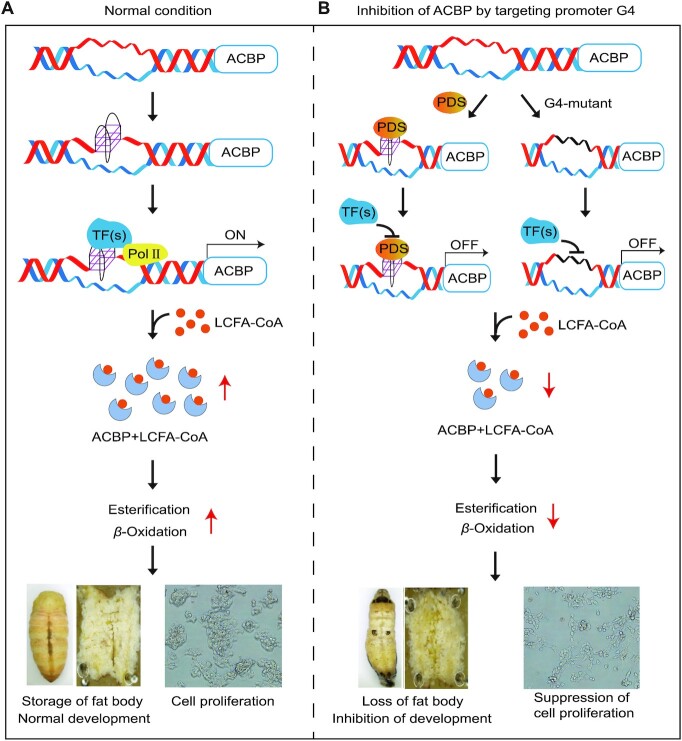
Diagram of the proposed mechanism of lipogenesis regulation by targeting of the G4 structure. (**A**) When *ACBP* gene starts to transcribe, the G4 structure forms and recruits transcription factors to turn on the transcription of *ACBP*. Then, ACBP proteins perform important functions in esterification and *β*-oxidation reaction. (**B**) The binding of transcription factors with G4 can be blocked by G4-specific binding compounds, such as PDS, or by mutation of the G4 structure by CRISPR–Cas9 to inhibit the expression of *ACBP* and result in reduced TAG levels and cell proliferation. Thus, the *ACBP* promoter G4 structure is a novel target for the development of insecticides and tumour-suppressive drugs.

G4 structures mediate the regulation of many biological activities by activating or suppressing relevant genes. Destruction of G4s in the promoters of the *col2a1*, *fzd5* and *nog3* genes in zebrafish embryos leads to reductions in the transcription of these three genes along with variations in growth phenotypes (10). Stabilized G4s in the promoter of human *c-myc* inhibit the transcription and translation of this gene, resulting in cell proliferation inhibition, cell cycle arrest and apoptosis (57). The present study revealed that a G4 structure formed in the *BmACBP* promoter and participated in the regulation of gene transcription processes, eventually affecting fat body and lipid metabolism. *ACBP* is essential in lipid metabolism processes, including ester synthesis and β-oxidation, because of its high binding affinity for acyl-CoA esters (19,28,58,59). ACBP, also known as the ‘hunger protein’, can stimulate appetite and induce lipogenesis (27). In the present study, we showed that a G4 structure can fold in the ACBP promoter of human HeLa, K562 and HepG2 cells (Figure 5A). The TAG levels were significantly reduced when the expression of HsACBP was inhibited by PDS treatment (Figure 5B and C). To further demonstrate the function of G4 in regulating lipogenesis, we directly mutated HsACBP G4 by CRISPR/Cas9. In the mutant cells, the TAG content was significantly decreased, and cell proliferation was suppressed (Figure 5D-L). Knocking out BmACBP resulted in a small larval body size, reduced fat body mass and lipid storage, and larval death at the 3rd instar stage (Supplementary Figure S4). In combination with the results of GO enrichment analysis of genes containing PQSs in their promoter regions, which showed many pathways of lipid metabolism, such as fatty acyl-CoA binding, the fat response, steroid hormone mediation and the steroid hormone receptor pathway (Supplementary Figure S2), we suggest that the ACBP G4 structure participates in the regulation of silkworm development, probably by regulating the lipid metabolism processes (Figure 6).

In summary, we found that G4 structures exist in the promoters of *ACBP* genes in invertebrates and vertebrates. Blocking the binding of transcription factors with G4 by PDS or mutating the G4 structure by CRISPR/Cas9 can inhibit the expression of ACBP and result in reduced TAG levels and cell proliferation (Figure 6). Thus, the *ACBP* promoter G4 structure is involved in regulating lipid metabolism and may be a novel target for the development of insecticides and tumour-suppressive drugs.

## DATA AVAILABILITY

Raw and processed data for CUT&Tag are available in the GEO repository under the accession number GSE201939. The other data that support the findings of this study are available from the authors on reasonable request.

## Supplementary Material

gkac527_Supplemental_Files
