# Supplementary material for: DNA G-quadruplex structure participates in regulation of lipid metabolism through acyl-CoA binding protein
Source: Nucleic Acids Res. 2022 Jun 24;50(12):6953–67. doi: 10.1093/nar/gkac527 (PMC9262599; doi:10.1093/nar/gkac527)
Supplement: gkac527_Supplemental_Files [file gkac527_supplemental_files.zip › Supplementary Data (NAR-00967-Q-2022).pdf]

SUPPLEMENTARY DATA

DNA G-quadruplex structure participates in regulation of lipid metabolism through Acyl-CoA binding protein

Lijun Xiang[1], Kangkang Niu[1,*], Yuling Peng[1], Xiaojuan Zhang[1], Xiaoyu Li[1], Ruoqi Ye[1], Guoxing Yu[1], Guojun Ye[1], Hui Xiang[1], Qisheng Song[2], Qili Feng[1,*]

[1]Guangzhou Key Laboratory of Insect Development Regulation and Application Research, Institute of Insect Science and Technology, School of Life Sciences, South China Normal University, Guangzhou 510631, China

[2]Division of Plant Sciences and Technology, University of Missouri, Columbia, MO 65211, USA

*To whom correspondence should be addressed. Tel: +86 20 85215291; Fax: +86 20 85215291; Email: qlfeng@scnu.edu.cn. Correspondence may also be addressed to Kangkang Niu. Tel: +86 20 85215291; Fax: +86 20 85215291; Email: kkniu@m.scnu.edu.cn.

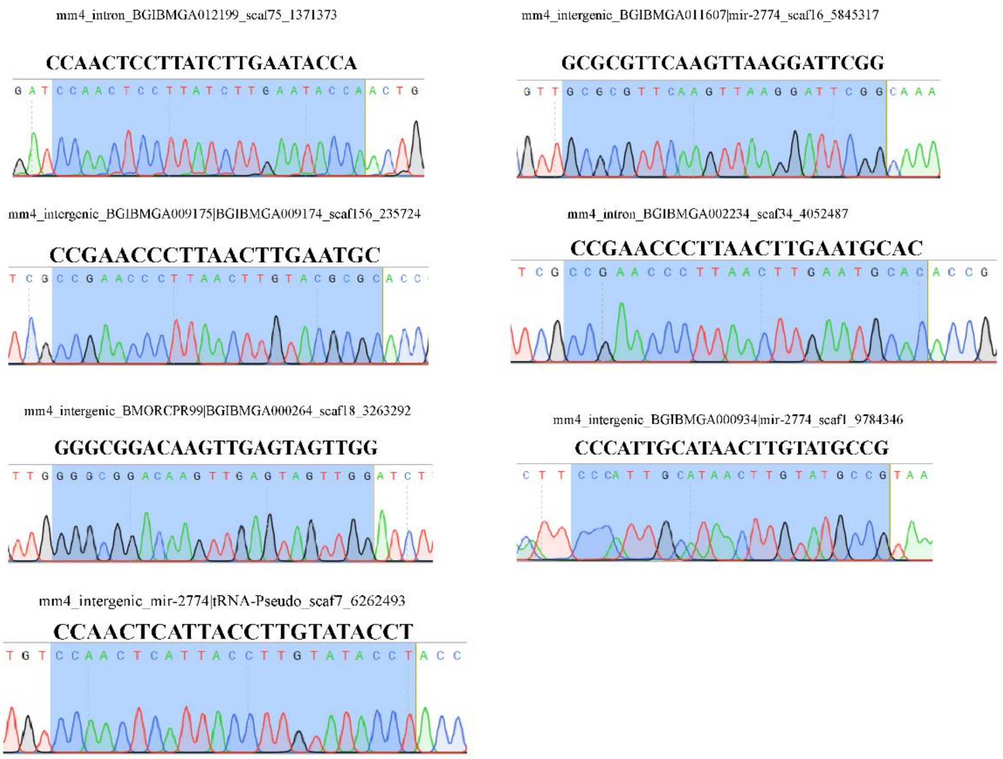

Supplementary Figure 1. The detection of off-target in *BmACBP* knock out experiment.

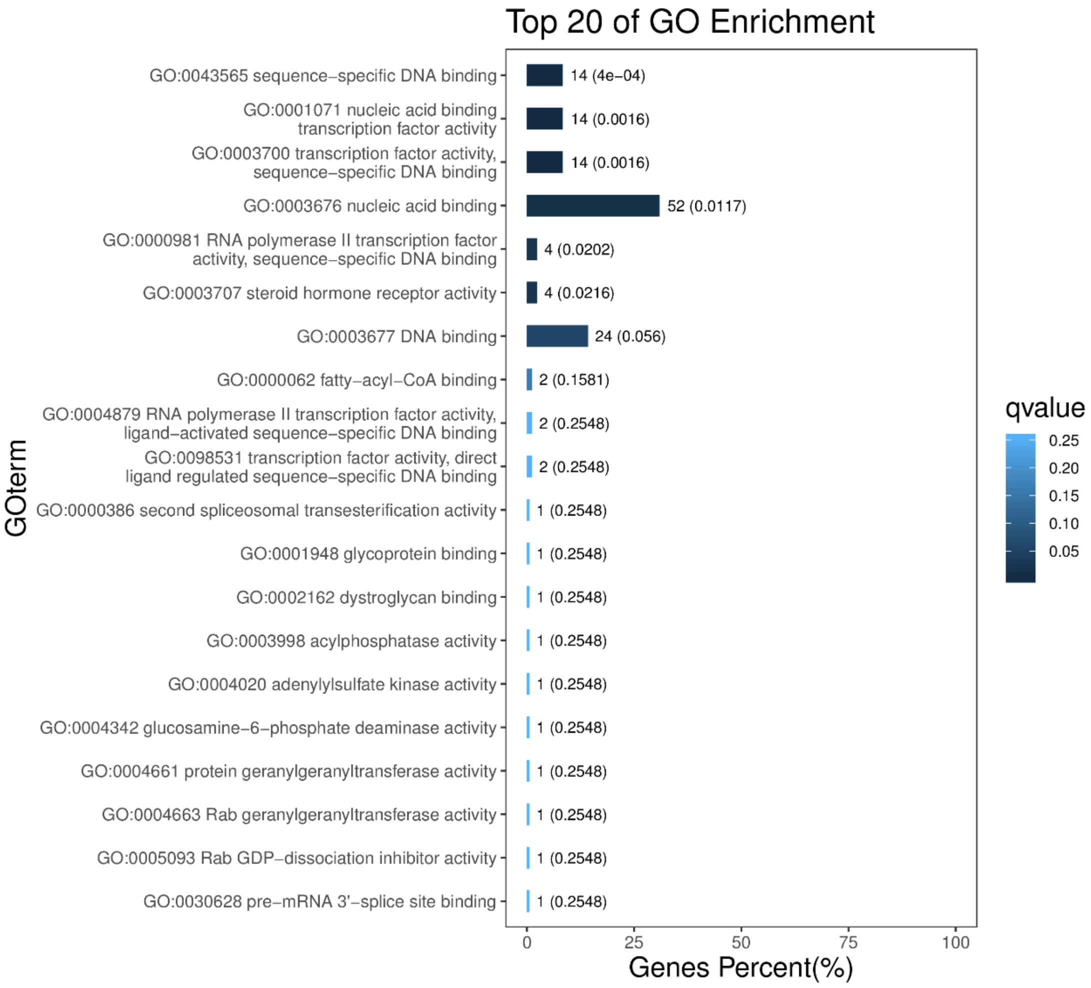

Supplementary Figure 2. The GO enrichment analysis of genes with G4 structure in 2 kb upstream of the ATG.

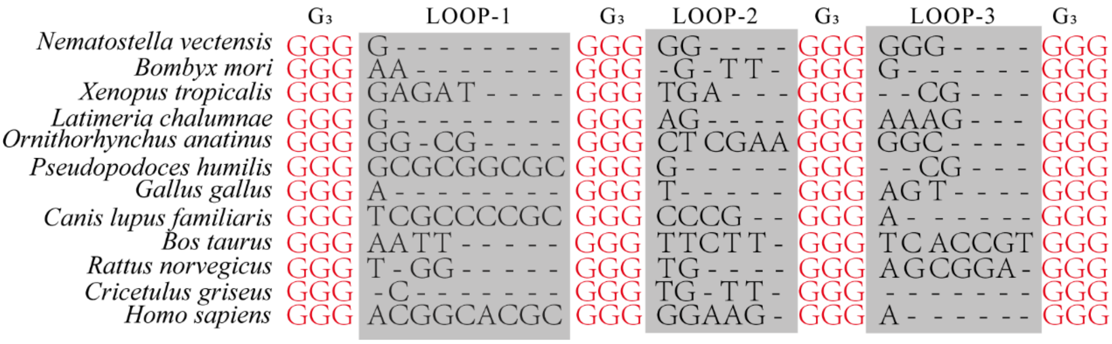

Supplementary Figure 3. Comparison of *ACBP* promoter G4 sequences in twelve species.

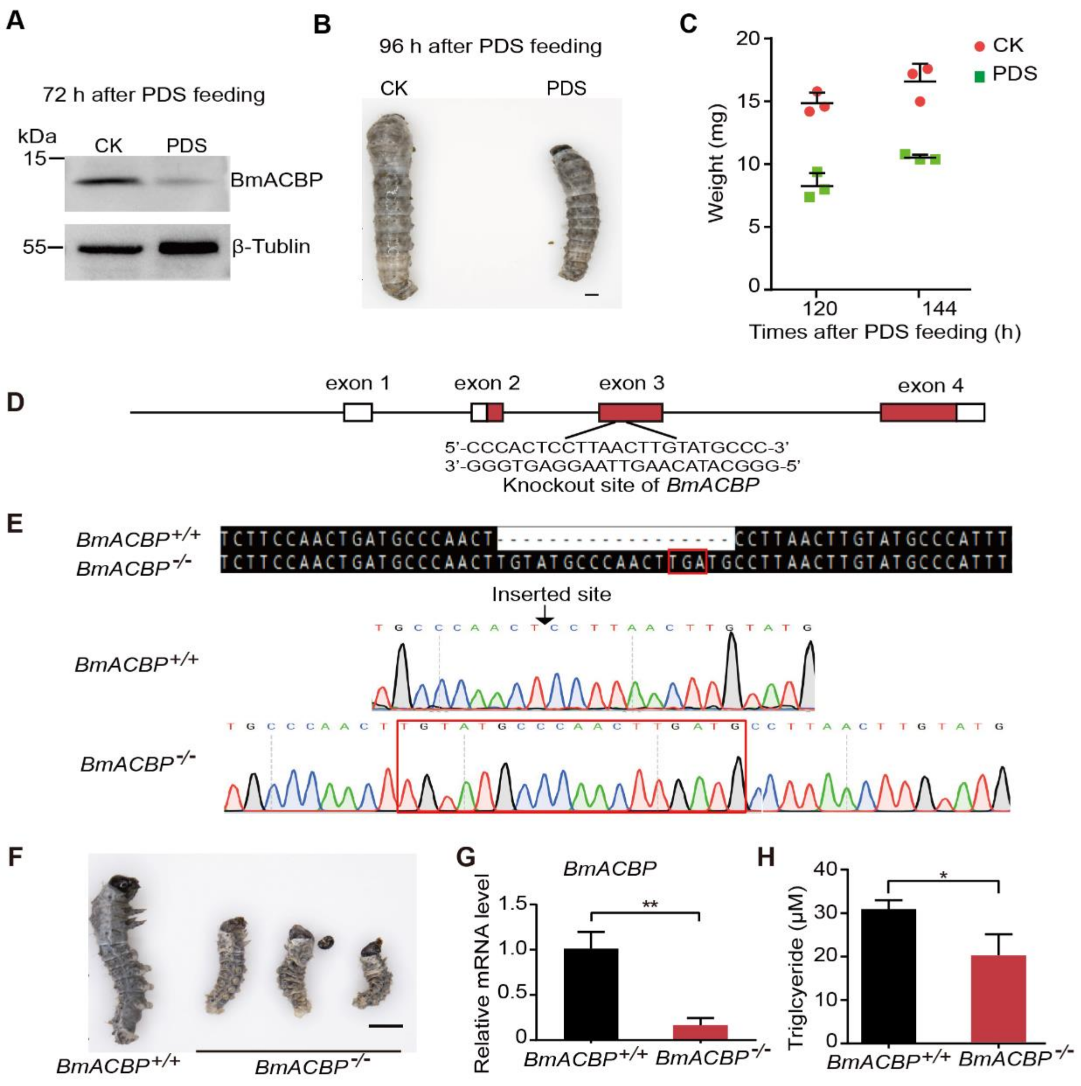

Supplementary Figure 4. Effect of PDS feeding and *BmACBP* knock-out on development of *B. mori*. (**A**) Western blot analysis confirming the BmACBP expression. (**B**) The morphological comparison between the experimental group and the control. The number of silkworms per treatment and control was 30. (**C**) The effect of PDS on body weight. The newly-hatched silkworms were fed with fodder supplemented with PDS for four days (see materials and methods). (**D**) The site designed for knock-out point. (**E**) Genomic sequencing analysis. (**F**) The comparison of phenotypes between the wild type and mutation. (**G**) The mRNA expression of *BmACBP* in wild type and mutation larvae. (**H**) Values for BmACBP$^{-/-}$ larvae triglyceride accumulation.

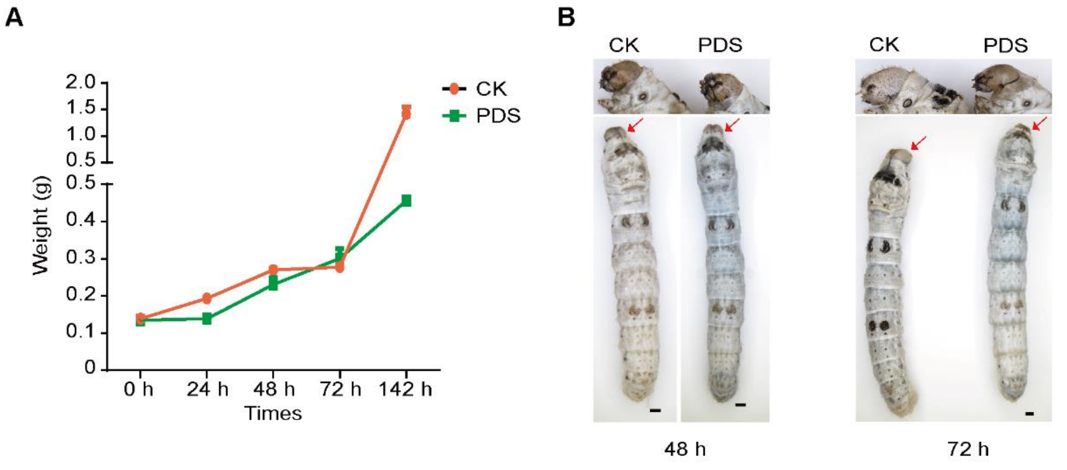

C

	72 h/5th instar larvae No. (%)	96 h/5th instar larvae No. (%)	120 h/5th instar larvae No. (%)	142 h/5th instar larvae No. (%)
CK	13 (68)	13 (100)	13 (100)	13 (100)
PDS	0 (0)	3 (37.5)	6 (75)	8 (100)

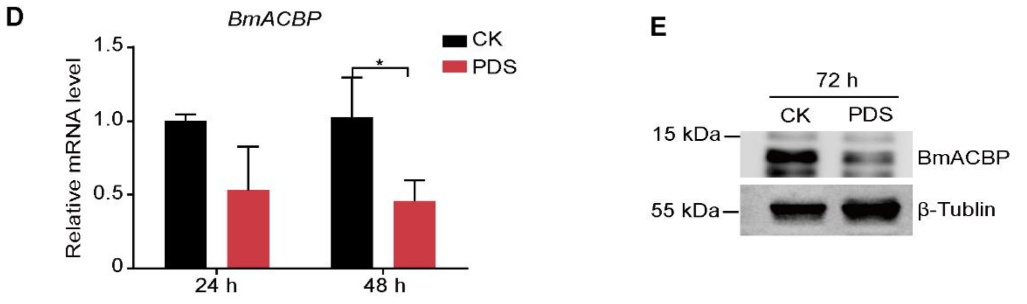

Supplementary Figure 5. Effect of PDS injection on development of 4th instar *B. mori*. (**A**) The effect of PDS on weight. (**B**) The morphological comparison between the experimental and control group 48 and 72 h after injection. (**C**) The number and proportion of larvae entering the 5th instar 72, 96, 120 and 142 h after injection. The number of silkworms per treatment and control was 30. (**D**) The mRNA expression of *BmACBP* after PDS injection in B. mori. (**E**) Western blot analysis showing positively decrease of BmACBP protein after PDS injection in *B. mori*.

Supplementary Table 1. The oligonucleotide used in this study.

Name	Sequence（5'-3'）	Used for
sg-BmACBP F	TAATACGACTCACTATAGGGCATACAAGTTAAGGAGTGTTTTAGAGCTAGAAATAGCAAGTTAAAATAAGGCTAGTCC	sg RNA template
sg-BmACBP R	AAAAGCACCGACTCGGTGCCACTTTTTCAAGTTGATAACGGACTAGCCTTATTTTAACTTGCTATTTCTAGCTCTAAAA	sg RNA template
KO-BmACBP F	GGACATTTTTATAGGAAACATTGG	KO test
KO-BmACBP R	CTACAATTGCTCTGCCCATA	KO test
HsACBP-g4ko-test-F	TCTTCAGGCCGGGCAAGATT	G4-KO test
HsACBP-g4ko-test-R	TTTAGAGGCGCGGTCCACTC	G4-KO test
BmACBP G4	GTCGGGAAGGGGTTGGGGGGGGGTAGGTT	*CD*
Mut BmACBP G4	GTCGTGAAGGTGTTGATGGAGTGTAGGTT	*CD*
HmACBP G4	TGGGGACGGCACGCGGGGGAAGGGGAGGGGA	*CD*
Mut HmACBP G4	TGCTGACGGCACGCGCTGGAAGCTGAGTGGA	*CD*
RatACBP G4	CGGGGTGGGGGTGGGGAGCGGAGGGGGA	*CD*
Mut RatACBP G4	CGGAGTGGATGTGACGAGCGGAGCTGGA	*CD*
CN hamsterACBP G4	CGGGTGTTGGGGGGTGTACTCGGGTTGAGGGC	*CD*
Mut CNhamster ACBP G4	CGCGTGTTGGGAGGTGTACTCGAGTTGAGTGC	*CD*
BmACBP-ChIP F	CTGAAGTCGGGAAGGGGTT	*ChIP*
BmACBP-ChIP R	AGTTTTGCCGGAAATCGAGAA	*ChIP*
FAM-Bm ACBP G4	FAM-GTCGGGAAGGGGTTGGGGGGGGGTAGGTT	*EMSA*
FAM-Mut BmACBP G4	FAM-GTCGTGAAGGTGTTGATGGAGTGTAGGTT	*EMSA*
FAM-ACBP G4 ligad	FAM-TACTCGACAGTAACCTACCCCC	*PCR-Stop*
BmACBP G4	GTCGGGAAGGGGTTGGGGGGGGGTAGGTT	*PCR-Stop*
BmACBP mut-G4	GTCGTGAAGATGTTGATAGGGGGTAGGTT	*PCR-Stop*
Q BmRP49 F	CAGGCGGTTCAAGGGTCAATAC	*qRT-PCR*
Q BmRP49 R	TACGGAATCCATTTGGGAGCAT	*qRT-PCR*
Q BmACBP F	GCCACAGTTGGAGATGCCGA	*qRT-PCR*
Q BmACBP R	GCCAGTTTGTGCCAAGCGTC	*qRT-PCR*
Q HsACBP F	GCCCGGGATGTTGGACTTCA	*qRT-PCR*
Q HsACBP R	AGCTTTCATGGCATCTTCCTTGG	*qRT-PCR*
BmACBP G4 ASO	AACCTACCCC CCCCCAACCC CTTCCCGAC	*ASO*
GFP-ASO	GCACAAGCTGGAGTACAACTACAACAG	*ASO Unspecific ssDNA*
FAM-Bm ACBP G4	FAM-GTCGGGAAGGGGTTGGGGGGGGGTAGGTT	*ASO*
FAM-ACBP-G4	FAM-GTCGGGAAGGGGTTGGGGGGGGGTAGGTT	*EMSA*
Q BmRP49 F	CAGGCGGTTCAAGGGTCAATAC	*qRT-PCR*
Q BmRP49 R	TACGGAATCCATTTGGGAGCAT	*qRT-PCR*
Q HsGAPDH-F	GAGTCAACGGATTTGGTCGT	*qRT-PCR*
Q HsGAPDH-R	GACAAGCTTCCCGTTCTCAG	*qRT-PCR*
Q BmACBP F	GCCACAGTTGGAGATGCCGA	*qRT-PCR*
Q BmACBP R	GCCAGTTTGTGCCAAGCGTC	*qRT-PCR*

Off-target-intron-F1	ACAGAACATTTCAGGGGCGA	*qRT-PCR*
Off-target-intron-R1	ACGAAGCCGAAATTAGCCGA	*qRT-PCR*
Off-target-intron-F2	GGCGGACTTAATGCCCAAGA	*qRT-PCR*
Off-target-intron-R2	AACCTCTCGAGATCAGGACT	*qRT-PCR*
Off-target-intergenic-F3	TTCCTCCGCTATTTGGTCCG	*qRT-PCR*
Off-target-intergenic-R3	ACCAAAGGTAGTCCAGAGTGG	*qRT-PCR*
Off-target-intergenic-F4	AGTCAGATTGCCGCCTTTGA	*qRT-PCR*
Off-target-intergenic-R4	CCTGCCGACCAGCGATATAG	*qRT-PCR*
Off-target-intergenic-F5	AATTTTCATACCCCGGGCCT	*qRT-PCR*
Off-target-intergenic-R5	CTGGAGCAACGCAAACAACA	*qRT-PCR*
Off-target-intergenic-F6	AGTACCAGCCACCAGTCAAC	*qRT-PCR*
Off-target-intergenic-R6	CGTAACGATAGAGACGCGGT	*qRT-PCR*
Off-target-intergenic-F7	CACGGCTGCAATCAAACCAA	*qRT-PCR*
Off-target-intergenic-R7	CGCGGATCGTAACATTTCGT	*qRT-PCR*
Off-target-intergenic-F8	TGCCGCCACATTGAAACATG	*qRT-PCR*
Off-target-intergenic-R8	ACCGGTTCACAAACATGGGA	*qRT-PCR*

Supplementary Table 2. The predicted possible off-target sites. The possible off-target sequences in bold.

mm4_intron_BGIBMGA012199_scaf75_1371373	CGCTAATACTTCCCAGTGACTTTTTTGTTTTCATACACGGACATATGAGAATGCGAT**CCAACTCCTTATCTTGAATACCA**ACTGGAGACATAAGTTCGATTTTGTTGTATAATAGGCTCCCCAATATATCAA
mm4_intergenic_BGIBMGA011607|mir-2774_scaf16_5845317	ACTCTGGCATCAGCTCACAATGATAATACACACAGAAATGACGTAAATCGAGATCAGGACTATTACTAACCGTT**GCGCGTTCAAGTTAAGGATTCGG**CAAATGTTATTGGAAAAGTTAGCGGAATCGTCATG
mm4_intergenic_BGIBMGA009175|BGIBMGA009174_scaf156_235724	TCATCTTAATAAGTTCATGACGATTCCGCTAACTTTTCCAACAACATTCG**CCGAACCCTTAACTTGTACGCGC**ACCGGTCAGCTTTAGTGCAAAGAATAAGGTTTCTTGAATTCAGAATCTAAGAAGTTCATAAA
mm4_intron_BGIBMGA002234_scaf34_4052487	GTTAACTTTTCCAACAGCATTCG**CCGAACCCTTAACTTGAATGCAC**ACCGGTTAGTAATAGTCCTGATCTCGAGAGGTT
mm4_intergenic_BMORCPR99|BGIBMGA000264_scaf18_3263292	TGCTAAACTTGTAAGAACATGTTG**GGGCGGACAAGTTGAGTAGTTGG**ATCTTTTTAAAGCCGAATTAGTTTATAAATTTTATGCTTATCATG

mm4_intergenic_BGIBMGA000934|mir-2774_scaf1_9784346	CACGGCTGCAATCAAACCAACTCTAATTAAGTCTGCATAATTCTCATAATGAACATT**CCCATTGCATAACTTGTATGCCG**TAAAGGCTAACTCGAGTCAACGAACTTTCGGGCTGTGACAAAAGTTAATTTCG
mm4_intergenic_mir-2774|tRNA-Pseudo_scaf7_6262493	AATAGTAAAAGCTAGTGACGGTGCCCAGATATCCAATTGT**CCAACTCATTACCTTGTATACCT**ACCTATATATATTTCGAAAATATTTTATTCAAATAGAATCTATTT

Dataset file 1. The background file of GO enrichment analysis.
